# Attomolar sensitivity microRNA detection using real-time digital microarrays

**DOI:** 10.1038/s41598-022-19912-z

**Published:** 2022-09-28

**Authors:** Fulya Ekiz Kanik, Iris Celebi, Derin Sevenler, Kahraman Tanriverdi, Nese Lortlar Ünlü, Jane E. Freedman, M. Selim Ünlü

**Affiliations:** 1 Department of Electrical and Computer Engineering, Boston University, Boston, MA USA; 2 Department of Biomedical Engineering, Boston University, Boston, MA USA; 3 Department of Surgery, Center for Engineering in Medicine, Harvard Medical School, Massachusetts General Hospital, Shriners Hospitals for Children, Boston, MA USA; 4 Division of Cardiovascular Medicine, Vanderbilt University Medical Center, Nashville, TN USA

**Keywords:** Biomarkers, Biomedical engineering, Optical techniques

## Abstract

MicroRNAs (miRNAs) are a family of noncoding, functional RNAs. With recent developments in molecular biology, miRNA detection has attracted significant interest, as hundreds of miRNAs and their expression levels have shown to be linked to various diseases such as infections, cardiovascular disorders and cancers. A powerful and high throughput tool for nucleic acid detection is the DNA microarray technology. However, conventional methods do not meet the demands in sensitivity and specificity, presenting significant challenges for the adaptation of miRNA detection for diagnostic applications. In this study, we developed a highly sensitive and multiplexed digital microarray using plasmonic gold nanorods as labels. For proof of concept studies, we conducted experiments with two miRNAs, miRNA-451a (miR-451) and miRNA-223-3p (miR-223). We demonstrated improvements in sensitivity in comparison to traditional end-point assays that employ capture on solid phase support, by implementing real-time tracking of the target molecules on the sensor surface. Particle tracking overcomes the sensitivity limitations for detection of low-abundance biomarkers in the presence of low-affinity but high-abundance background molecules, where endpoint assays fall short. The absolute lowest measured concentration was 100 aM. The measured detection limit being well above the blank samples, we performed theoretical calculations for an extrapolated limit of detection (LOD). The dynamic tracking improved the extrapolated LODs from femtomolar range to $\sim$ 10 attomolar (less than 1300 copies in 0.2 ml of sample) for both miRNAs and the total incubation time was decreased from 5 h to 35 min.

## Introduction

Among nucleic acids, molecules essential to all known forms of life, RNAs have an important role in understanding the nature of cellular processes as they function as transmitters for genetic information in cells, and regulate transcriptional and post-transcriptional processes^1^, hence they can serve as biomarkers for monitoring cellular activity. Most common RNA biomarkers are messenger RNAs (mRNAs) and non-coding RNAs such as microRNAs (miRNAs). Recent advances have enabled the discovery of these nucleic biomarkers, and their potential in clinical applications such as cancer and neurodegenerative disease detection^2, 3^. Although conventional nucleic acid detection technologies have been adequate for mRNA detection, sensitive miRNA detection, especially in multiplexed format, has remained a challenge due to its relatively short sequence length, low abundance and sequence similarity within families of biomarker miRNAs^4, 5^.

MicroRNAs are small, non-coding RNA molecules containing $\sim$ 18–25 nucleotides that regulate post-transcriptional factors of gene expression by binding directly to the target mRNA to alter protein translation or interacting directly with expressed proteins^6, 7^. Recently, it has been shown that not only the presence/absence of a gene but also minor fluctuations in expression levels of a particular gene can also be indicative for a disease and its stage^8, 9^. Therefore, miRNA quantification becomes crucial to track minute changes in gene regulation. Increased or decreased levels of specific miRNAs have been shown to play a significant role in pathological processes and currently, more than 1900 human miRNAs are reported to be linked to various diseases^10^. These nucleic acids have been found to be linked with viral^11, 12^ and bacterial^13^ infections, as well as non-infectious diseases such as cardiovascular disorders^14^ and cancer^15^. Consequently, miRNA detection has become an emerging field in diagnostics and prognostics, providing information about pathology staging and aiding with clinical treatment guidance^16, 17^.

Since miRNAs are present at very low copy numbers in a cell, from a few to several hundred only^9^, and abundance per sample volume is very small in clinical samples^18^, the technique for detecting and quantifying miRNAs must be very sensitive to be clinically relevant. Traditional techniques have been unable to adequately meet the need for multiplexed detection at sub-femtomolar concentration. One of the earliest methods of detection, northern blotting, although considered the gold standard, cannot distinguish miRNAs with different sequences and require relatively large volumes of sample^19^. Popular amplification-based methods such as reverse transcription-quantitative polymerase chain reaction (RT-qPCR), and digital droplet PCR (ddPCR) can offer quantitative results. However, due to the short length of miRNAs, base pairing may be mismatched during amplification causing reduced selective PCR efficiency and therefore false positives^20, 21^. Furthermore, these methods require high purity samples, advanced technological equipment, high capacity infrastructure, and personnel expertise for operation and analysis of the clinically relevant information. Microarray technology has also been applied to miRNA research^22^ to provide high throughput and multiplexing. However, conventional microarray technologies have limited dynamic range and sensitivity, and also suffer from specificity issues^21, 23^. Previous studies in the literature using DNA assays similar to our approach have demonstrated the capabilities of this technology, however failed to realize the full potential due to the limitations of analog measurement modalities^24^. We employ single molecule counting, which inherently is a digital measurement method, to overcome the limits of analog detection^25^. We demonstrate accurate detection of zeptomoles of target particles present on the surface.

Previously, single molecule counting capability of single-particle interferometric reflectance imaging sensor (SP-IRIS) was demonstrated to identify protein biomarkers on antibody assays^26^. Here, we describe the development of a DNA assay for real-time enumeration of individual microRNAs with attomolar sensitivity on a multiplexed digital microarray platform, utilizing SP-IRIS. Interferometric reflectance imaging provides optical detection of target nanoparticles captured onto a simple reflecting substrate, with single-molecule sensitivity^27, 28^. SP-IRIS nucleic acid assays are conceptually very similar to conventional DNA microarrays; however, SP-IRIS assays provide digital counting of captured molecules, in contrast to conventional platforms. Using plasmonic gold nanorods (GNRs) as labels to tag individual nucleic acid targets, coupled with polarized illumination to increase visibility of the nanoparticle labels, this platform allows low numerical aperture (NA) optics, hence greater field-of-view (FOV) and in effect, increased throughput and sensitivity^29^. Moreover, in contrast to leading single-molecule detecting technologies that are built upon endpoint assays, SP-IRIS can provide kinetic measurements by dynamic particle tracking^30^. Kinetic assays with single-molecule readout measure directly the individual capture of target molecules in real-time, further improving the limit of detection (LOD). Therefore, dynamic particle tracking is an excellent solution for specifically detecting low-abundance biomarkers in the presence of low-affinity but high-abundance background molecules, where endpoint assays fail.

We present experimental results for two fairly abundant miRNAs, miR-451 (microRNA-451) and miR-223 (microRNA-223-3p), for our proof-of-concept assay development applications. MiRNA-451 plays a role in tumorigenesis, tumor suppression, self-renewal and chemo-resistance in many types of cancers, such as breast, gastric and colorectal, and has been implicated as a diagnostic and prognostic biomarker^31^. MiR-223 expression plays a role in influenza or hepatitis B infections, inflammatory bowel disease, type 2 diabetes, leukemia and lymphoma and upregulated in recurrent ovarian cancer^32^.

We compare endpoint and kinetic measurements where we utilize dynamic tracking of target particles and establish miRNA detection with $\sim$ 10 attomolar sensitivity. We obtain a highly sensitive and multiplexed digital microarray for two types of miRNAs, demonstrating the potential of this assay with optimized capture agents, for a robust, ultra-sensitive and selective diagnostic platform.

## Methods

### SP-IRIS chips and fabrication of microarrays

The SP-IRIS chips are fabricated by thermally growing an oxide layer on a polished silicon substrate, followed by photolithographic patterning of the oxide layer. The microarrays are created by spatially separated immobilization of different DNA probes on the sensor surface.

An ideal surface for probe immobilization should have reactive functional groups enabling covalent binding of the probes, minimal non-specific interaction with the medium and, chemical stability against environmental changes throughout the experiments. For a proper surface chemistry and efficient surface probe immobilization, we selected an anti-fouling and functional polymer for microarray surface modification. The IRIS chip surface is coated with MCP (copoly-DMA-MAPS-NAS) polymers from Lucidant Polymers (Sunnyvale, CA, USA). This polymer is commonly used to coat glass, silicon, or other hydroxylated surfaces for microarray applications described in detail elsewhere^33^. The MCP polymers consist of three different monomers: dimethylacrylamide (DMA) enables self-adsorption to the oxide substrate, 3-(trimethoxysilyl) propyl methacrylate (MAPS) provides covalent binding to the substrate through silane functional groups, and acryloyloxysuccinimide (NAS) presents NHS ester groups which are utilized in biomolecule probe covalent immobilization. In this study we have used MCP-2, MCP-2F which provides more hydrophobic surfaces preventing spreading of the spots, and a mixture of the two copolymers (MCP-2 containing 5% MCP-2F) which we will refer to as 5% MCP-2F. See Supplementary Fig. S1 for a detailed structure of the polymers. The polymers were shown to have increased probe density compared to 2D coatings involving silane chemistry and also very low non-specific binding^34^. Yalcin et al.^35^ have shown that the MCP polymer swells 7–20 nm in liquid solution, which elevates the immobilized probe molecules that are already attached to the polymer and makes them more available for surface reactions.

To prepare the surface prior to polymer coating, the chips were cleaned by sonicating in acetone, rinsed with isopropanol and DI water and dried under nitrogen. They were functionalized prior to spotting by submerging the chips for 30 min in the copolymer solution, rinsing with DI water, drying under nitrogen and eliminating excess moisture by placing the chips in an 80 $^{\circ }$C oven for 15 min. Complementary and control surface probes were spotted on the IRIS chips in an array format using Scienion sciFLEXARRAYER S3 piezoelectric robotic arrayer. Single stranded DNA (ssDNA) surface probes were spotted at a concentration of 20 $\upmu$M in 150 mM phosphate buffer. A microarray pattern was designed for sixteen replicate spots for each DNA. The space between the spots (pitch) was set to 200 $\upmu$m.

After spotting, the DNA-spotted chips were kept in the spotter at 67% humidity overnight at room temperature to allow time for the amine-modified ssDNA surface probes to immobilize on the polymer surface. Following the immobilization, the chips were incubated in blocking solution of 0.1 M Tris buffer with 50 mM ethanolamine (pH 8.5) for 1 h to quench the excessive NHS groups left on the polymer after immobilization. The chips were then washed with DI water. The spotted chips were kept in a desiccator under vacuum until further use.

### DNA probe design

Synthetic miRNAs and custom-designed ssDNA oligonucleotide surface probes were purchased from Integrated DNA Technologies (IDT) (Coralville, IA). Table 1 shows the sequences for miR-451 and miR-223, and their complementary surface probes. The surface probes were amine-functionalized (“AmMC6”) for covalent binding to the copolymer coated IRIS chips, while GNR label sequence was thiol-modified (“ThioMC6”) for nanorod conjugation, as provided by IDT. The surface probes also have a double-stranded “stabilizer” region between the surface and the miRNA complementary region to accelerate hybridization with the target sequence.

**Table 1:** Sequences for miRNAs and their complementary surface probes.

**Name, Type**	**Sequence**
miR-451a, RNA	AAACCGUUACCAUUACUGAGUU
miR-223-3p, RNA	UGUCAGUUUGUCAAAUACCCCA
miR-451a	AACTCAGTAATGGTAACGGTTTTAGGACTAGGACTTGAAGTTGAAGTTTT/3AmMC6T/
Surface Probe, DNA	
miR-223-3p	TGGGGTATTTGACAAACTGACATAGGACTAGGACTTGAAGTTGAAGTTTT/3AmMC6T/
Surface Probe, DNA	
Non-complementary	/5AmMC6/ ATATGTACCCACCGCTATTCCAGTCTGTTCATTCGTAGGC
Surface Probe, DNA	
Surface Probe Stabilizer	CTTCAACTTCAAGTCCTAGTCCTA
Sequence, DNA	
GNR label	5ThioMC6-D/TTTTTTTTTTTTTTTTT
Sequence, DNA	

### GNR poly-T conjugation

GNR-polyT conjugates were prepared with slight modifications to previously described methods^36, 37^. Prior to conjugation, thiol-modified polyT GNR label oligonucleotides were treated with DTT to cleave the disulfide bonds and to activate thiols. 20 $\upmu$M thiol-polyT was prepared in 170 mM phosphate buffer pH 8.0 and mixed with 100 mM DTT. The solution was kept in a dark at room temperature for 3 h while occasionally vortexing. PolyT oligos were then desalted and purified using NAP-5 column according to the manufacturer’s directions. Meanwhile, 6 mL of nanorods (OD1, approximately 10 pM) were centrifuged at 1500 rcf for 15 min and resuspended with DI water in a glass container. Immediately after obtaining polyT oligos from the column, the purified oligonucleotides were quantified by measuring their absorbance at 260 nm using a NanoDrop spectrophotometer. GNRs and label oligos were mixed in the glass container and placed on an orbital shaker for 30 min. Then, to bring the pH of the solution to pH 3, a concentrated citrate buffer solution was added up to a final concentration of 10 mM citrate. Similarly, Tween-20 was added to have a final concentration of 0.1% w/v. After vortexing and sonicating, the solution was rested on the orbital shaker over-night. In the following 24 h, the salt concentration of the solution was brought up to 150 mM by slowly adding citrate buffer with high concentration of NaCl. Salt addition was followed with another overnight resting. Finally, the nanorods were washed three times with PBS with 0.1% Tween-20 to remove the excess, unbound oligonucleotides. The conjugation was confirmed by spectrophotometric analysis. The GNR-polyT conjugate was kept in PBST (phosphate buffer saline with 0.05% Tween-20) without direct light, for up to 2 months at room temperature for further use. Before every use, the conjugate was washed once again to remove the surfactant.

### Polyadenylation of miRNAs

Poly-A tails were added to the synthetic RNA sequences with the commonly used polymerase enzyme (poly-A polymerase, PAP). This method can be used for synthetic or natural RNA molecules in a pure solution or in complex media, due to its robustness^38^. PAP continuously adheres free adenosine nucleotides in the solution to the free 3’ ends of RNA molecules. The active reaction time was 5 min at 37 $\,^{\circ }$C, followed by a high temperature destruction of the enzyme activity at 56 $\,^{\circ }$C for 10 min. The reaction time yields around a 30 long poly-A tail formation.

### Reagents and apparatus

Dithiothreitol (DTT) was obtained from Sigma-Aldrich (Woodlands, TX, USA). NAP-5 columns for DNA purification were purchased GE Healthcare. Citrate-capped nanorods (OD1) with a longitudinal surface plasmon resonance peak at 650 nm and nominal dimensions 25 nm by 71 nm were used as plasmonic labels (Nanopartz Inc, Loveland, CO, part number A12- 25-650-CIT). MCP-2 and MCP-2F copolymers were purchased from Lucidant Polymers (Sunnyvale, CA, USA) and used for chip surface functionalization. All other chemicals were purchased from Sigma-Aldrich. NanoDrop 2000c Spectrophotometer from Thermo Fisher Scientific (Waltham, MA) was used for characterization of nanoparticles. Scienion S3 SCIFLEXARRAYER (Berlin, Germany) spotter was used to create an array of DNA surface probes. Hamilton PHD 2000 syringe pump was used in liquid experiments.

### Image acquisition, particle detection and dynamic tracking

**Figure 1:**
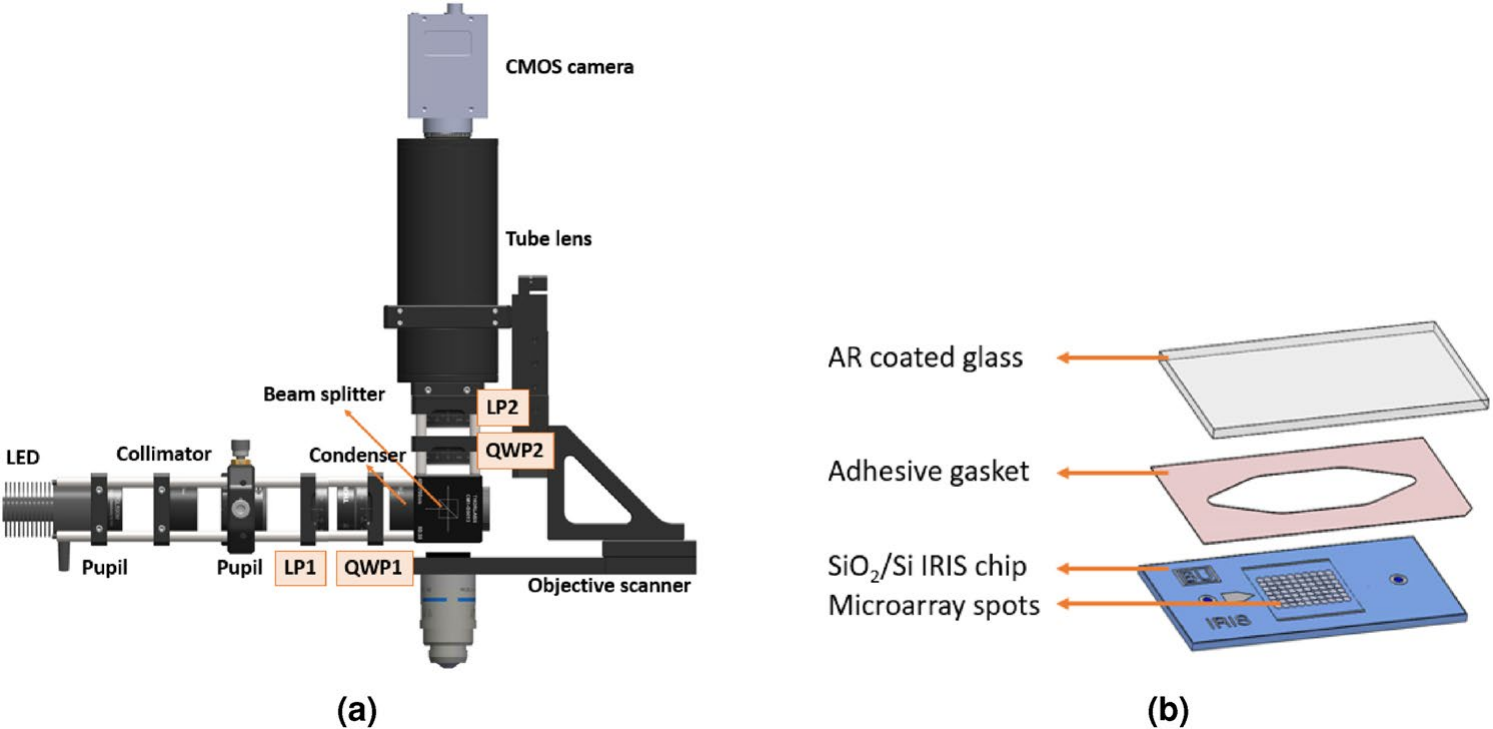
Optical setup of the polarization enhanced SP-IRIS (**a**) and the SP-IRIS cartridge assembly (**b**). To form the fluidic chamber, the gasket is sandwiched between the chip and the AR coated glass.

The experiments were performed in both heterogeneous assay format where end point measurements were taken, and homogeneous assay format where real-time images were taken in liquid for dynamic tracking of particles with a 1.1-inch format CMOS GS3-U3-123S6M-C (FLIR) and a 20x/0.45 NA NIKON objective with correction collar. Figure 1 shows the imaging setup (a), and the SP-IRIS fluidic cartridge (b) that is composed of the prepared chip, an adhesive spacer and an AR coated glass to form the flow chamber, used for in liquid constant flow experiments.

Custom plug-ins were used for image acquisition through the open-source microscope control application, Micromanager. Temporal averaging was optimized to reduce shot noise in the system and a sparse pseudomedian filter, with a kernel selected to be slightly larger than the image point spread function, is used to smooth the image. The image acquisition employs an objective scanner to obtain z-stacks, in order to observe defocus curves while selectively attenuating the reference field by the polarized collection path^29^.

After acquisition, a custom software, SPANDEX, is used for particle detection and tracking. With the custom software, the images are first normalized by the median values of each pixel from the z-stack acquired, then, the difference between the maximum and minimum grayscale values at each (x,y) pixel position is determined. After applying a threshold, each visible dot location in the differential image is designated as a particle. To eliminate false negatives and false positives, nanoparticles in the image are detected by (1) performing a cross-correlation with a measured image kernel, (2) binarization of the image with a predetermined global threshold, and (3) filtering of key-points based on size and area-to-perimeter ratio to include only diffraction limited spots. Keypoint filtering thresholds were iteratively optimized manually once and then applied identically to all images. For the dynamic tracking of particles, a post-processing algorithm is applied where the cumulative number of binding events is recorded. The new binding and debinding events are detected by observing particle presence on the (x,y) location that was previously assigned for a particle or at a new (x,y) location. With a prior knowledge of dwell times of the targets, the false decisions are eliminated by repairing gaps between frames (false negative) or removing single frame positives from the count^30^. All image acquisition and processing software described here has been made freely available online at https://github.com/derinsevenler/spandex.

#### Polarization-enhanced single-particle reflectance imaging sensor (SP-IRIS)

The SP-IRIS instrument is a reflection based high-resolution wide-field microscope that utilizes Kohler illumination with an LED source. The visibility of individual particles that are smaller than the illumination wavelength is increased with two enhancements: (i) optimizing the thickness of the thermally grown oxide layer for maximum interference signal and (ii) utilization of polarized illumination. The thickness of the oxide film (110 nm) on the sensor is optimized for the specified plasmonic label dimensions (25–71 nm) by performing a BEM simulation in MATLAB. To exploit the unique scattering properties of the plasmonic rods, we utilize circularly polarized illumination where the reference field can be selectively attenuated. The GNRs only scatter the excitation component that is polarized along its longitudinal axis, at its resonance wavelength (650 nm), therefore the scattered light is a function of angle between incident light and GNR’s longitudinal axis^29^. By changing the focus position of the objective, all particles are made visible regardless of their orientation.

**Figure 2:**
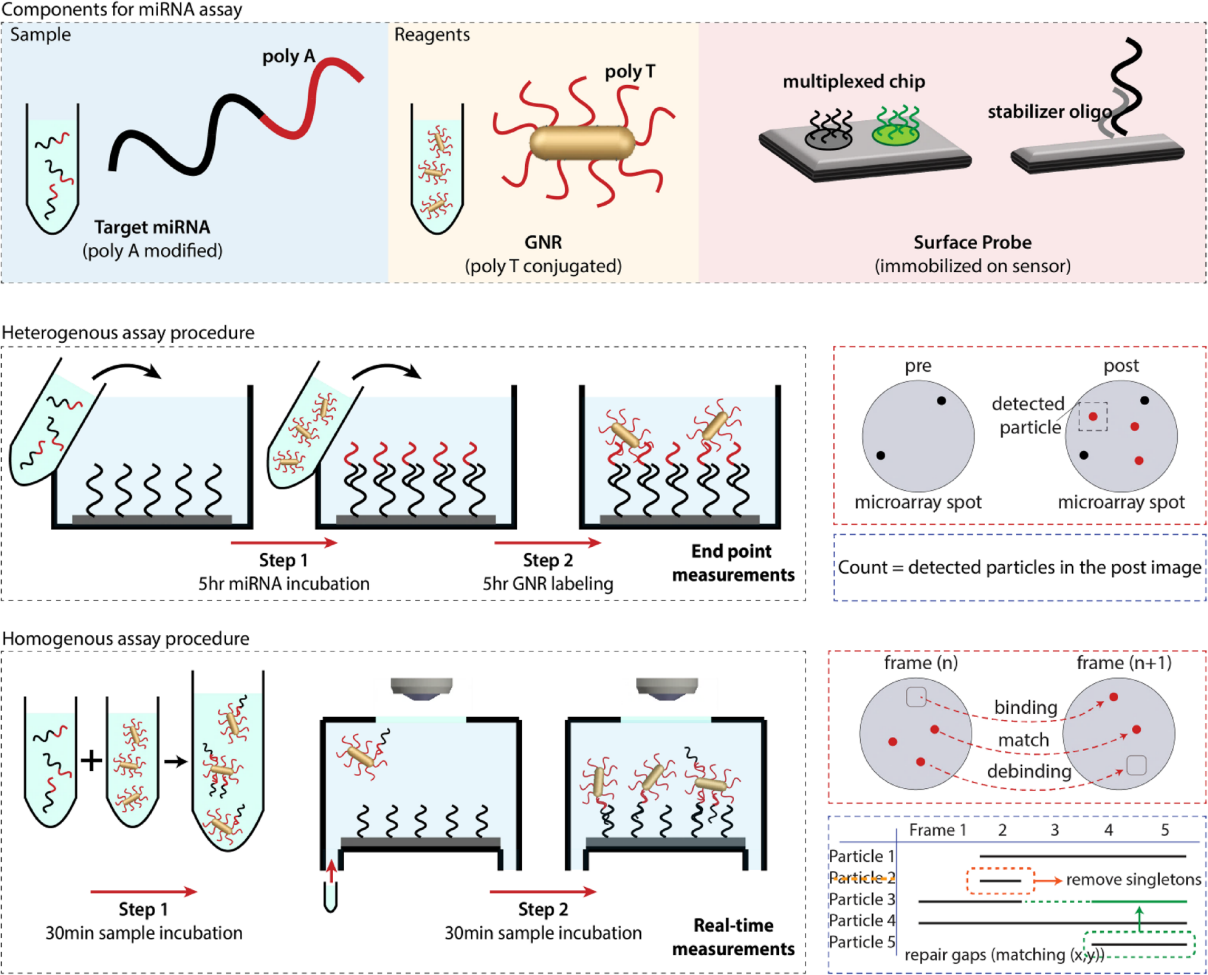
Components for the miRNA assay and experimental steps of heterogeneous and homogeneous assay procedures.

### Experimental procedure

Synthetic miRNA samples were polyadenylated, a process widely used in literature^24, 39^ by using PolyA polymerase in order to add polyA tails to the 5’ end. PolyT-conjugated GNRs were used for labeling through polyA ends on miRNAs. Polymer coated chips were preincubated with the stabilizer sequence in order to lift the surface probes in solution and accelerate hybridization. The chips were incubated with 1 $\upmu$M of stabilizer oligo in 2xSSC buffer for 20 min. After incubation, the chips were washed with 2xSSC for 2 min and 0.2xSSC for 2 min, and dried.

MiRNA detection was done in heterogeneous and homogeneous assay formats. In heterogeneous assay, the microarray was incubated with the miRNA sample for 5 h to capture the target on the functionalized surface, then placed in the microfluidic chamber and incubated with polyT-conjugated GNRs for another 5 h at room temperature. After rinsing the microarray with buffer solution for 10 min, endpoint images were taken. Particle counting was done using SPANDEX custom software to obtain total particle counts. Individual particles bound to target miRNAs were identified and digitally counted. In homogeneous assay, first, miRNA samples and polyT-conjugated GNRs were mixed in a vial before incubation. Then, the mixture was injected into the microfluidic chamber with the microarray chip and a real-time video of the surface was captured during 1 h incubation at a flow rate of 5 $\upmu$L/min. The video was used for to dynamically track the individual binding/debinding events of miRNAs, which are labeled with polyT-conjugated GNRs on the microarray. Figure 2 shows the schematic representation of two types of assay procedures.

## Results

### Optimization for assay conditions

**Figure 3:**
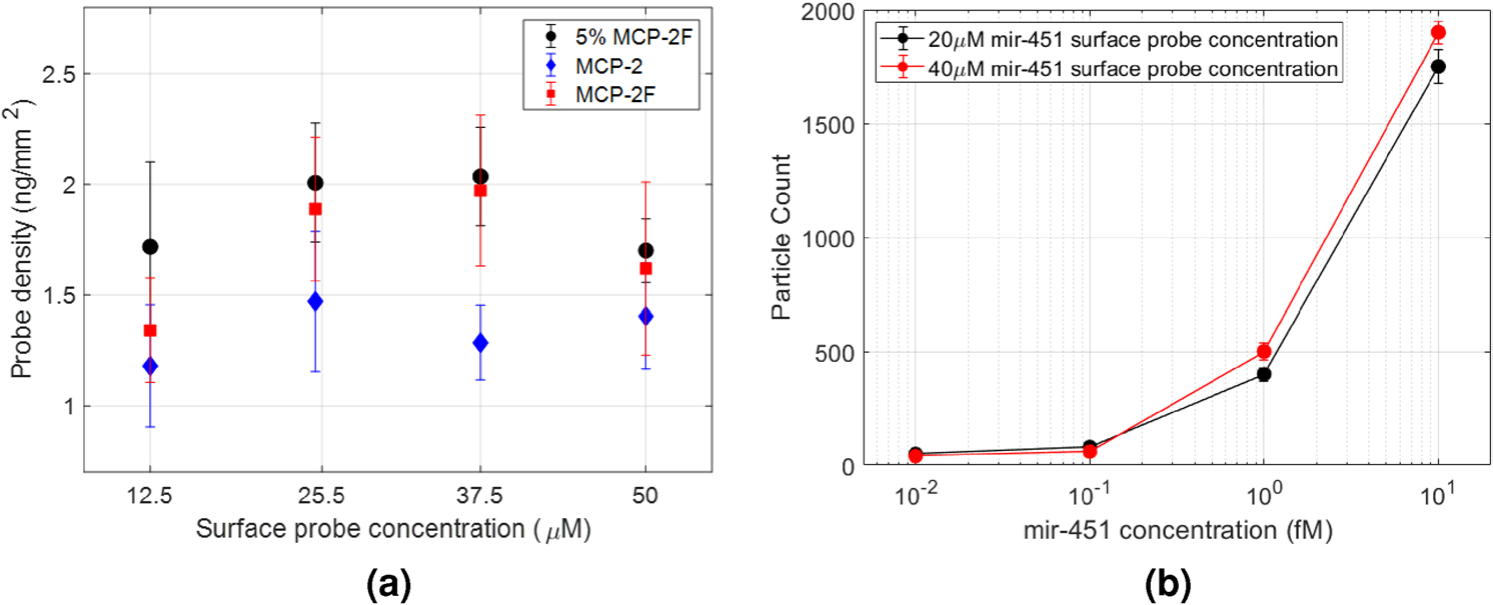
Comparison of immobilized surface density with respect to three different polymers at different probe spotting concentrations (**a**). Effect of probe spotting concentration in particle capture efficiency (**b**).

As described in methods, the MCP polymers are ideal for immobilizing the surface probes and enabling them for biomolecule capture. Two copolymers, MCP-2 and MCP-2F, and their mixture, 5% MCP-2F, were tested in this study. They differ in ratio of the monomers and the pendant moieties. MCP-2F copolymer provides a more hydrophobic surface with its lower surface energy which prevents spreading and merging of the spots. The oligonucleotide surface probe for miR-451 was spotted on the different polymer coated chips at 12.5 $\upmu$M, 25 $\upmu$M, 37.5 $\upmu$M and 50 $\upmu$M concentrations. Surface morphology and probe densities were compared. All three polymers enabled homogeneous DNA spots with excellent spot morphology. The probe height and density were analyzed using IRIS^40^, a low magnification modality of the SP-IRIS where the reflectance image is converted to corresponding surface thickness. Figure 3a shows probe densities as a function of the immobilization concentrations of surface probes on three polymer surfaces. MCP-2F surface has the smallest spots with a diameter of 65 $\upmu$m due to its hydrophobicity and reduced amount of probe immobilization. On the other hand, MCP-2 polymer provides the largest spots with an average diameter of 130 $\upmu$m. The microarray spots held by the 5% MCP-2F solution maintained a smaller average diameter of 90 $\upmu$m while preserving perfect spot morphology with its optimal hydrophobicity. Since MCP-2 and 5% MCP-2F polymers have similar immobilization efficiencies, and we prefer to fit as many spots as possible in a FOV for multiplexing experiments, we selected 5% MCP-2F copolymer for the miRNA studies.

DNA surface probe density is crucial for efficient target hybridization. The negative charge from neighboring DNA strands on the same spot can hinder hybridization efficiency. Hence, high probe density may not necessarily provide higher binding in hybridization assays. To observe the effect of probe density, four arrays were spotted with 20 $\upmu$M and 40 $\upmu$M concentrations of the surface probe for miR-451 (Figure 3b). These spots had similar levels of probe thicknesses, resulting in an average density of 2 ng$/\mathrm{mm}^2$. The arrays were incubated with four concentrations of synthetic miR-451 target and labeled with polyT-conjugated GNRs. After incubation, the chips were washed and imaged with SP-IRIS. This result suggests that the surface reaches to a saturation with 20 $\upmu$M probe density. Increasing probe concentration in spotting does not result in higher binding or hybridization efficiency even if the probe density is increased. Therefore, spotting concentration was chosen to be 20 $\upmu$M for the optimum surface probe density in array preparations.

#### Heterogeneous SP-IRIS miRNA assay

Although in this study, the focus is to demonstrate the sensitivity of homogeneous flow experiments and real-time tracking, we performed the heterogeneous end point experiments for comparative enhancement of detection limits.

In heterogeneous experiments, it was observed that longer incubation time for miRNA samples resulted in more captured nanoparticles. Incubation times (with a sample volume of   2 mL) of 2 h, 3 h, 4 h and 5 h were tested for both miRNA sample and polyT-conjugated GNRs. The best results were obtained with 5 h miRNA incubation and 5 h GNR-polyT labeling. Supplementary Fig. S2 shows the average particle counts registered against concentration (16 replicate spots each) after 5 h target incubation followed by 5 h GNR label incubation. Using the double logarithmic response plots we have shown the correlation between the particle counts and target concentration. The calibration curve (Supplementary Fig. S2) implies a power law dependence on the target concentration which is a softer dependence than the theoretical expectation, however this is a common observation in the literature^5^.

The limit of detection (LOD) was calculated using the linear trend, and the mean and standard deviation of blank samples (3.3 $\sigma$/slope, $\sigma$ = standard deviation of blank, n = 3) to set the limits as done in the previous studies^41, 42^. Dynamic ranges were 1 fM to 1 pM and 100 aM to 1 pM for miR-223 and miR-451, respectively. LOD was calculated as 2.22 fM for miR-223, and 228 aM for miR-451.

### Homogeneous SP-IRIS miRNA assay

Homogeneous assay configuration has a simple protocol with a single incubation step. The miRNA sample was pre-mixed with polyT-conjugated GNRs in a vial for 30 min, at room temperature. The microarray chip was then incubated with the pre-mixed solution. Compared to binding on solid phase support (heterogeneous format), a much more rapid response was observed in the real-time homogeneous assays. Incubation time for the miRNA-GNR complex in the flow chamber was below 60 min and in most cases response recorded under 30 min was sufficient to observe particle binding rates.

**Figure 4:**
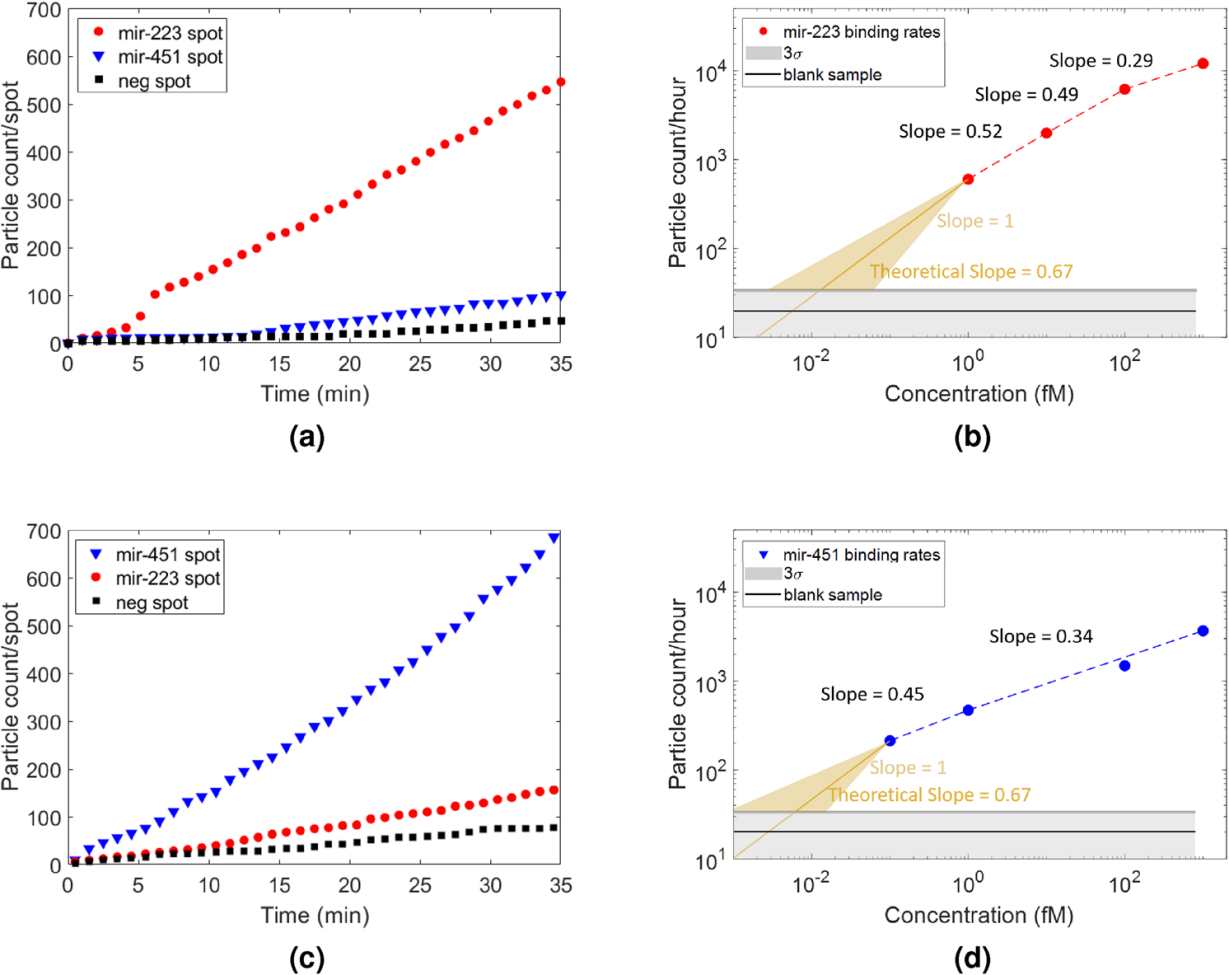
Cumulative number of binding events on each spot type throughout the homogeneous incubation with 10 fM miR-223 target complex and 100 fM miR-451 target complex (**a**,**c**). Binding rates throughout the homogeneous assay incubation with respect to target concentration (**b**,**d**).

Since we are able to detect and count every single binding event on the relevant spot throughout the experiment, we can track binding and debinding of each miRNA-GNR. Here, we represent total binding, meaning that when a miRNA-GNR complex binds to its complementary surface probe and debinds at equilibrium, we still include this complex for detection and quantification. We focus on total binding event count rather than particle counts on the surface of the sensor in every frame for increasing the sensitivity. Figure 4 shows the results for registered counting of cumulative (total) binding events of the miRNAs to the target and off-target spots in the same FOV versus time at one selected concentration for each miRNA target and binding rates against the concentration range.

During the kinetic experiments, a total of 12 spots were imaged for target, off-target and non-complementary control spots simultaneously during the course of incubation. Figure 4a and c show cumulative binding events on separate chips under kinetic incubation with 10 fM miR-223 target complex and 100 fM miR-451 target complex, respectively. No significant binding on the non-complementary control spots was observed. MiR-451 has shown some cross-reactivity towards off-target miR-223 spots. Same specificity issue was also observed in heterogeneous assays yet, it was improved in homogeneous assays where we observed less non-specific binding to off-target spots.

To demonstrate the response rates, standard curves were prepared with a range of analyte concentrations between 100 aM and 1 pM, shown in Fig. 4b and d. The target particle binding rates (particle count/hour) for each concentration were extracted from the binding curves (see Supplementary Figs. S3 and S4).

We have previously shown that the binding rate depends on the sheet density of target particles (which is proportional to square of cube root of volumetric concentration, $\propto c^{2/3}$) when the sample concentration is low (refer to Supplementary Figs. S5, S6, S10–13 for supporting studies and a more detailed explanation). In Figure 4b and d the particle counts per hour and the linear trends on the double-log plot approaching the theoretical slope (0.67 = $\Delta c^{2/3}$ ) are demonstrated as the concentration decreases. Here the LODs were calculated as the intersection of the theoretical linear trend and 3 standard deviations above the mean of blank sample. We also highlight the interval for the LOD calculation, lower bound being the extrapolation of the linear best fit on the double-log plot, upper bound being the extrapolation from the steepest possible slope. The interval yields a range from 1 aM to 50 aM. The conservative approach yields etrapolated LOD levels at 6.2 aM for miR-451 and 13 aM for miR-223. Hence, we obtained two orders of magnitude improvement in sensitivity of our microarray with dynamic tracking and assay duration was dropped from 5 h to 35 min. Directly measuring probe-analyte interactions during the course of the incubation allows for counting total individual binding events throughout the assay, distinguishing between specific and non-specific binding and obtaining binding rates for different samples. These advantages of kinetic assays with single-molecule detection enabled a lower LOD and reduced assay time significantly. To validate the detection limits of the system we have demonstrated functionalized gold sphere detection on an antibody surface with $5\times 10^3$ particle/ml (approximately 6 aM) concentration (Fig. S7 of the Supplementary Information). In these experiments, counts clearly above the background can be obtained within minutes or only 100 s (sub-attomoles) of analytes presented to the sensor. A comparison among the current miRNA detection assays reported in literature is given in Table 2.

**Table 2:** Comparison of current miRNA detection techniques.

Method	Matrix	miRNA	Dynamic range	LOD	Assay duration
Competition assay based on fluorescence quenching of Au NPs^43^	Aldehyde-activated glass slide	miRNA-205	3.8 pM–10 nM	3.8 pM	7 h hybridization
Surface plasmon resonance imaging (SPRI)^24^	LNA array	miRNA-16 miRNA-122b miRNA-23b	10 fM–1 pM	10 fM	4 h hybridization
Photonic Resonator Absorption Microscopy: Au NP SPR coupled photonic crystal (PC) guided resonance^5^	PC-adhered polydimethylsiloxan	miRNA-375 miRNA-1290	100 aM–1 pM	100 aM	2 h hybridization
Electrochemical biosensor based on poly(U) polymerase mediated isothermal signal amplification^44^	Electrochemically deposited AuNPs on Au surface	miRNA-319a	10–1000 fM	1.7 fM*	2 h hybridization, 1.5 h reaction time
Electrochemical biosensor based on analyte triggered nanoparticle localization and hybridization chain reaction dual amplification^45^	Tetrahedral DNA with the recognition hairpin immobilized on a gold electrode	miRNA-17-5p	100 aM–100 pM	2 aM*	2 h hybridization
Fluorescence-based Single Molecule Array (Simoa)^46^	Capture probes on microbeads	miRNA-16 miRNA-21 miRNA-141	1–1000 fM	0.68 fM* 1.60 fM* 0.58 fM*	2 h hybridization
Digital detection with real-time polarization enhanced SP-IRIS (this study)	MCP2-2F (5%) Copolymer	miRNA-451 miRNA-223	**100 aM–1 pM** **1 fM–1 pM**	6.2 aM* 13 aM*	**0.5 h hybridization**

*Denotes the calculated LOD levels by extrapolation below the lowest measured concentration.

Significant values in this study are in bold.

#### Multiplexed detection

In order to test multiplexing capability of SP-IRIS miRNA microarray, two of the miRNAs were mixed together prior to the experiment. 10 fM miR-223 sample and 100 fM miR-451 sample were mixed together with GNRs and incubated for 30 min. We followed the same protocol described previously, for the rest of the experiments.

**Figure 5:**
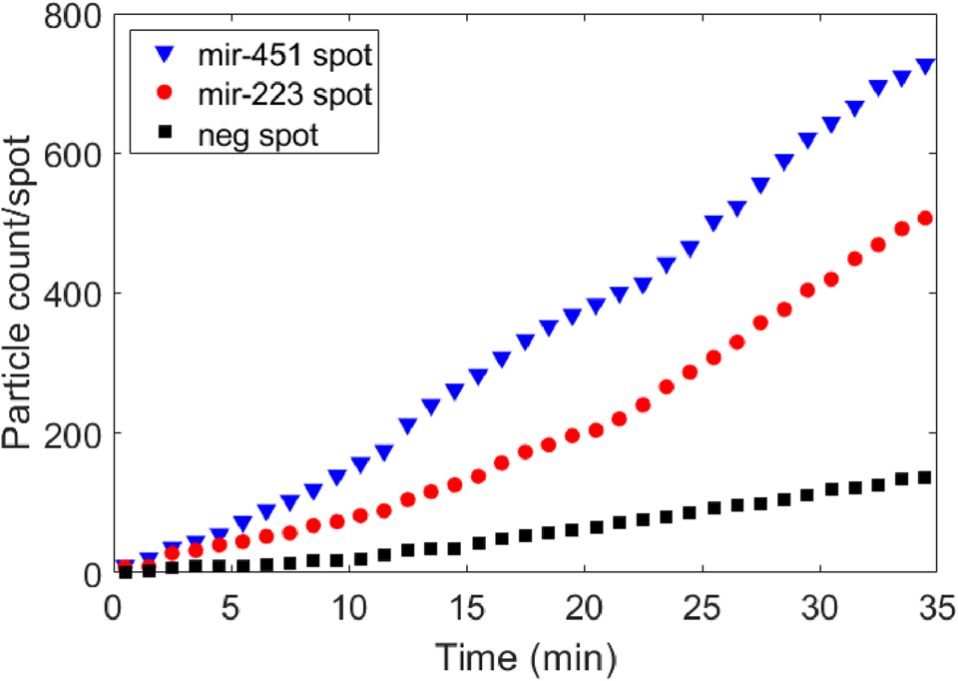
Cumulative number of binding events on each spot type throughout the incubation in a multiplexed format.

The two separate spots dedicated to miR-451 and miR-223 detection were imaged in the same FOV, identical to previous procedures. We observed excellent binding on the two spots simultaneously. Figure 5 shows the cumulative binding events after applying the tracking algorithm. We have observed slight changes of the total particle counts for the targets, which may be due to some specificity issues.

## Discussion

DNA microarray technology is a powerful tool for numerous applications including gene expression analyses and a wide range of end point assay techniques have been developed for single molecule read-out for detection of target nucleic acid sequences. However, sensitivity and specificity limitations have hindered the potential for clinical applications of nucleic acid detection on DNA microarrays, particularly due to its low-abundance when low-affinity but high-abundance background molecules are present. Introducing dynamic measurements to overcome these challenges of single molecule detection is essential for developing a clinically relevant diagnostic tool to track minute changes in gene expression that are linked with infections, non-infectious diseases and cancer. In this study, we developed an assay with plasmonic GNRs as labels for miR-223 and miR-451, for the proof of concept analysis of detection and quantification of miRNAs and we have successfully shown the advantages of dynamic tracking. In this study our dynamic range upper bound is 1 pM using the suggested assay time. The lower bound however, can be well below the attomolar level if the assay time was adjusted to increase the probability of sparse particles interacting with the surface within the flow cell.

We have demonstrated attomolar sensitivity for detection of individual miRNAs on SP-IRIS platform, enabled by real-time single particle counting with a digital DNA microarray. The lowest measured concentration of the miRNAs was 100 aM, yielding a binding rate well above the blank sample. Hence the following LOD levels were extrapolated. The limit of detection was improved from 2.22 fM to 13 aM for miR-223 and 228 aM to 6.2 aM for miR-451 when we implemented the dynamic measurements. Moreover, total assay time was reduced one order of magnitude which is critical for a diagnostic tool platform. With the proof-of-concept experiments, we have shown the potential of this platform, enabling digital detection of miRNA biomarkers in a multiplexed format.

## Supplementary Information

Supplementary Information.

## Data Availability

The RNA sequences used during the current study are available in the miRBase repository. Hsa-miR-223-3p accession: MIMAT0000280. Hsa-miR-451a accession: MIMAT0001631. Complementary sequences were created accordingly (IDT OligoAnalyzer Tool).
