# Supplementary material for: Attomolar sensitivity microRNA detection using real-time digital microarrays
Source: Sci Rep. 2022 Sep 28;12:16220. doi: 10.1038/s41598-022-19912-z (PMC9519543; doi:10.1038/s41598-022-19912-z)
Supplement: Supplementary file 1 — Supplementary Information. [file 41598_2022_19912_MOESM1_ESM.pdf]

Attomolar sensitivity microRNA detection using real-time digital microarrays

Fulya Ekiz Kanik[1], Iris Celebi[1], Derin Sevenler[1, 3] Kahraman Tanriverdi[4], Nese Lortlar Ünlü[2], Jane E. Freedman[4], and M. Selim Ünlü[1, 2*]

1 Department of Electrical and Computer Engineering Boston University, Boston, MA, USA

2 Department of Biomedical Engineering, Boston University, Boston, MA, USA

3 Department of Surgery, Center for Engineering in Medicine, Massachusetts General Hospital, Harvard Medical School, and Shriners Hospitals for Children, Boston, MA, USA

4 Division of Cardiovascular Medicine, Vanderbilt University Medical Center, Nashville, TN, USA

Supplementary Information

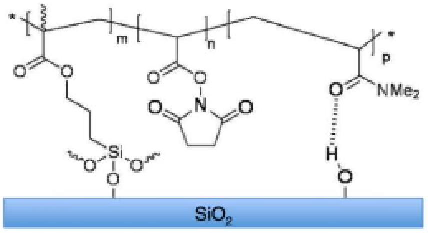

(a)

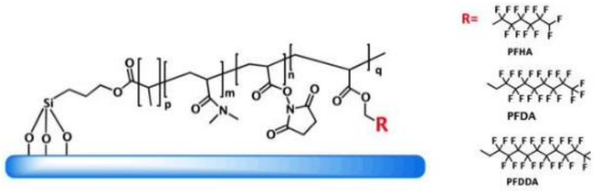

(b)

Figure S1. Chemical structure of the Lucidant (DMA-NAS-MAPS) copolymer [1] (a), MCP-2F [2] (b) coated on a glass surface.

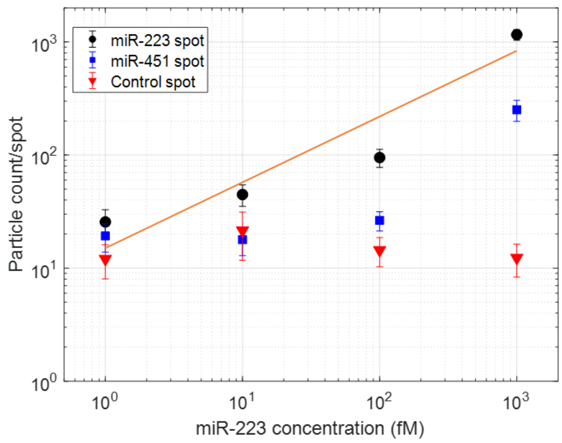

(a)

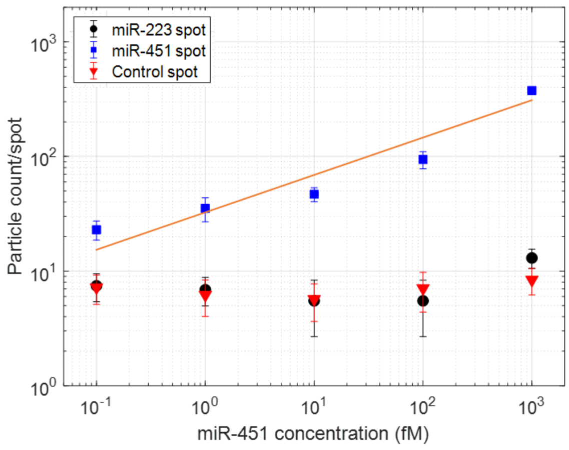

(b)

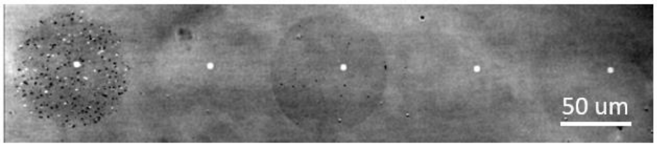

(c)

Figure S2. Average particle counts on spots with surface probes for miR-223 and miR-451 after incubation with miR-223 (a), and miR-451 (b). Image of particle accumulation (left spot) on the chip (c).

In Figure S2, the average nanoparticle counts (for n=16 replicate spots on one chip) are plotted against miRNA concentration for two miRNAs after incubation with miR-223 (a) and, miR-451 (b) for 5h following with 5h GNR label incubation in heterogeneous sandwich assay regime. The double logarithmic response plots show the correlation for miR-223 and miR-451 microarrays between the particle counts and target concentration. Figure S2 (c) shows an example image of the sensor, from the end point measurement.

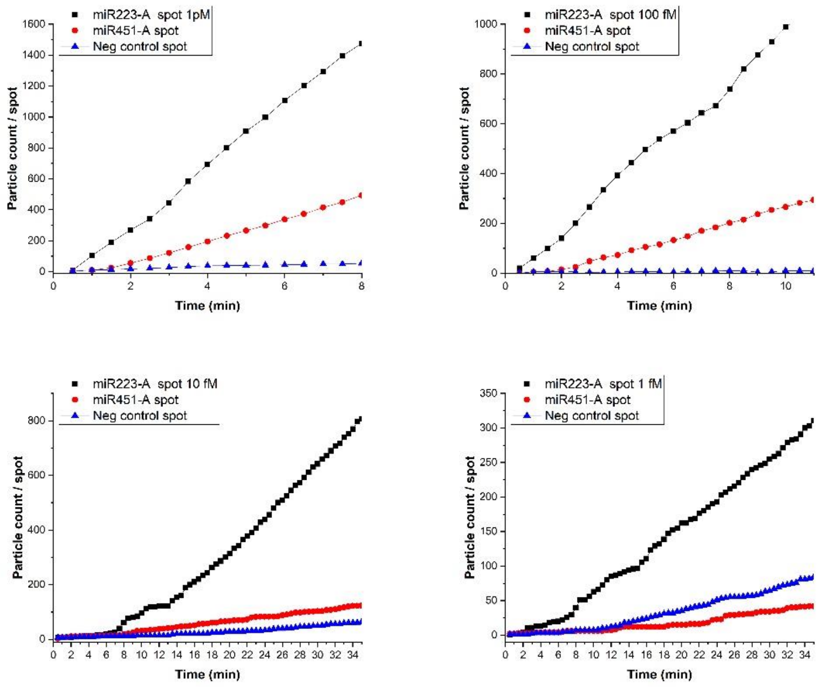

Figure S3. Mir-223 target flow: Particle counts on one example spot, shown for concentrations of 1pM, 100fM, 10fM and 1fM

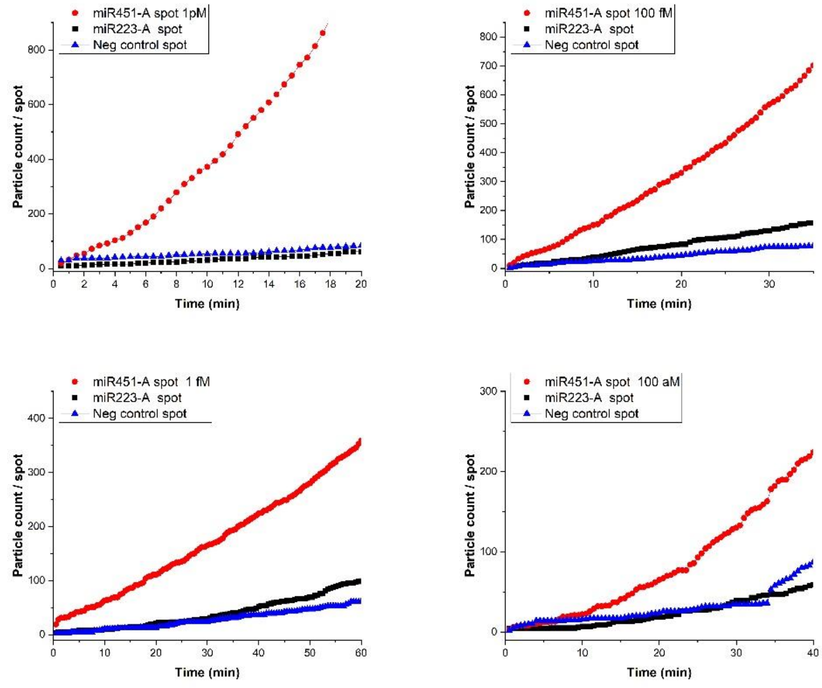

Figure S4. Mir-451 target flow: Particle counts on one example spot, shown for concentrations of 1pM, 100fM, 1fM and 100aM

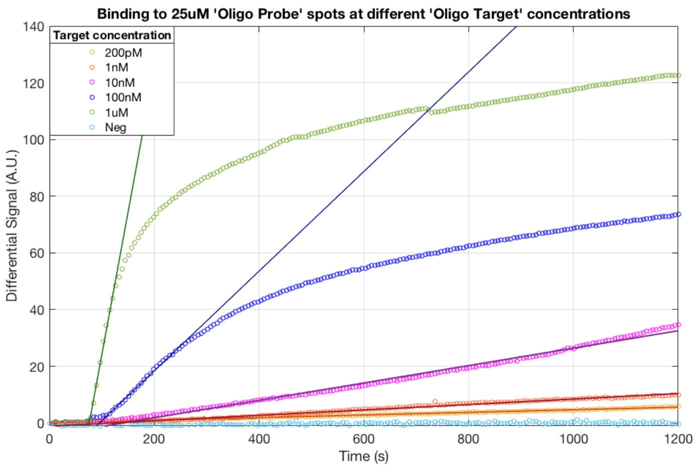

Figure S5. Binding signal extracted from an IRIS chip spotted with the oligonucleotide sequence complementary to the target solution. The binding experiment was performed with different target concentrations.

Figure S5 shows the binding curves on an IRIS chip functionalized with a commercial MCP-2 polymer (Lucidant). The chip surface was spotted with probes at 25uM, with a complementary sequence to the 40bp long target oligonucleotides. During the experimental procedure solutions at different concentrations of the target oligos were flown (200µl/min), with PBS and NanoPure water washes in between to restore the surface. We observe that the binding is linear in the beginning, then when the binding sites on the surface start to saturate, the binding rate slows down due to steric hindrance for high concentrations. Lower concentrations show that the binding remains always linear in time since not as many binding sites are occupied for steric hindrance to play a significant role.

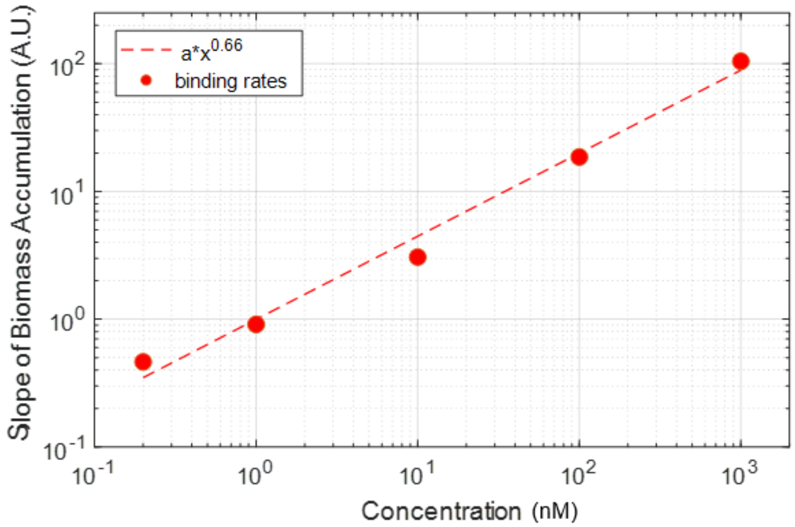

Figure S6. Extracted binding rates of experiments with different target concentrations and an exponential curve fitting ($a*x^{0.66}$) with an R-square of 0.987.

Figure S6 shows the extracted binding rates from the linear regions of the curves. We expect the binding levels to be proportional to the sheet density of the target solution across the surface, which is proportional to the square of the cube root of the concentration. Based on this theory we have demonstrated an exponential fit (linear fit in the double-log graph) with a high goodness of fit.

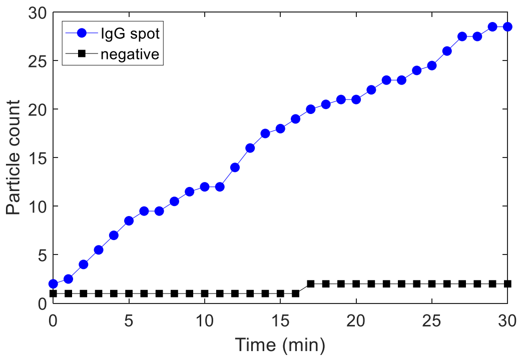

(a)

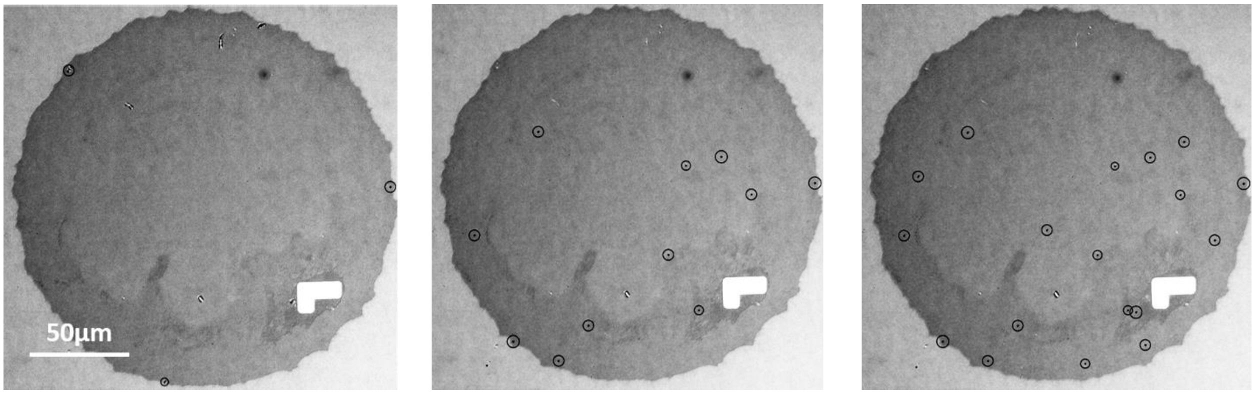

(b)

Figure S7. Example IgG spot and detected particles from the pre image, after 10min and 20min of flow of GNPs

A set of capture experiments were carried out with functionalized gold nano particles and antibody microarray surfaces to validate the limit of detection level of the system with a proof-of-concept set up. The 80nm Au particles are conjugated with Goat Anti Human IgG (Nanopartz) and the IRIS chip is functionalized with IgG spots. Direct capture of the target particles were recorded for different dilutions ($5x10^9$, $5x10^7$, $5x10^5$, $5x10^3$ #/ml) during 30min of flow with 50µL/min. Figure S7 shows the total particle count registered from $5x10^3$ #/ml target particle concentration, averaging (n=3) IgG spots and the background of the same effective area to serve as the negative region. The lowest concentration in this experiment ($5x10^3$ #/ml) translates to approximately 6 aM. Figure S7 (b) shows an example spot from this flow experiment.

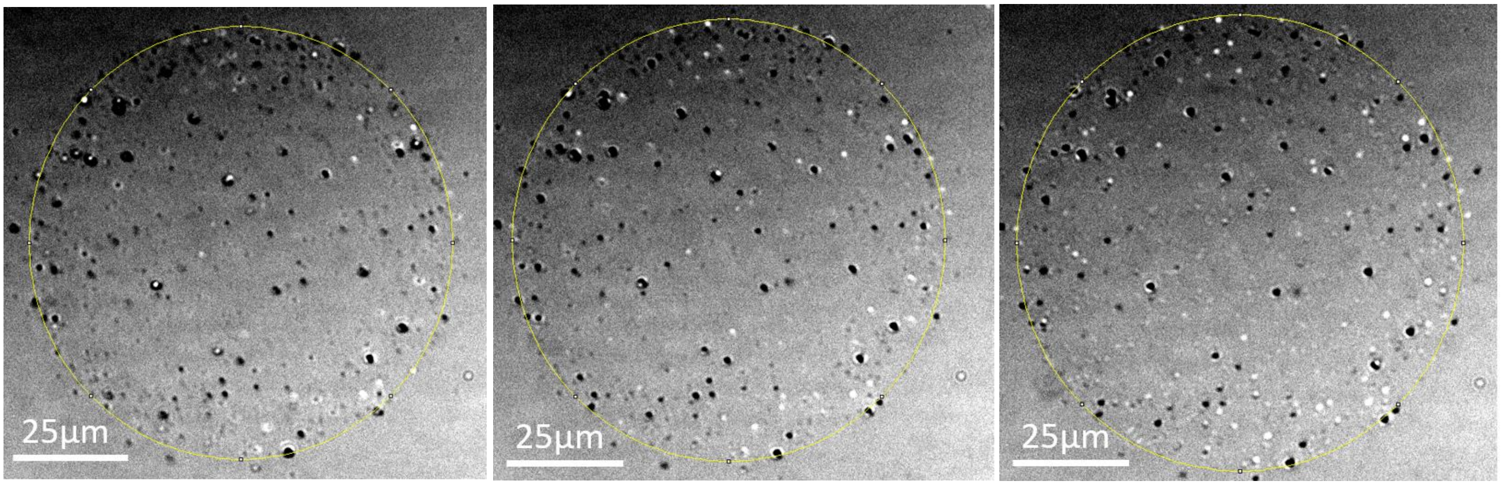

(a) (b) (c)

Figure S8. Raw images of spots with captured target nucleic acids, tagged with nanorod labels. The focal position difference in the optical path is 1µm apart.

Figure S8 shows the raw images taken from a single time point in the real-time movie (homogeneous assay, miRNA-451 spot with 100fM target concentration), at different focal positions. The stacks are converted into normalized intensity images, processed and passed through the detection algorithm. Sevenler et al [4], explains how the nanorods are made visible, with minimized orientation dependency, utilizing circularly polarized illumination in the SP-IRIS system.

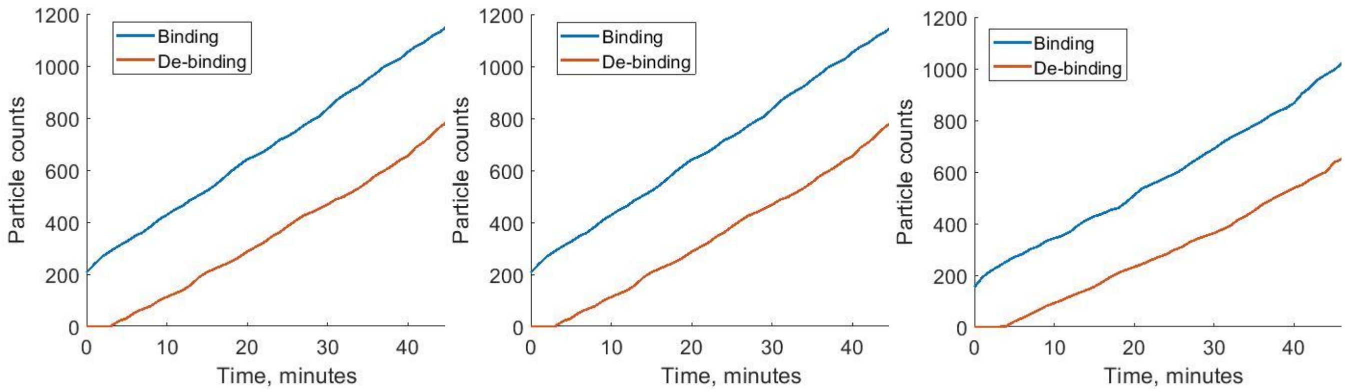

(a)

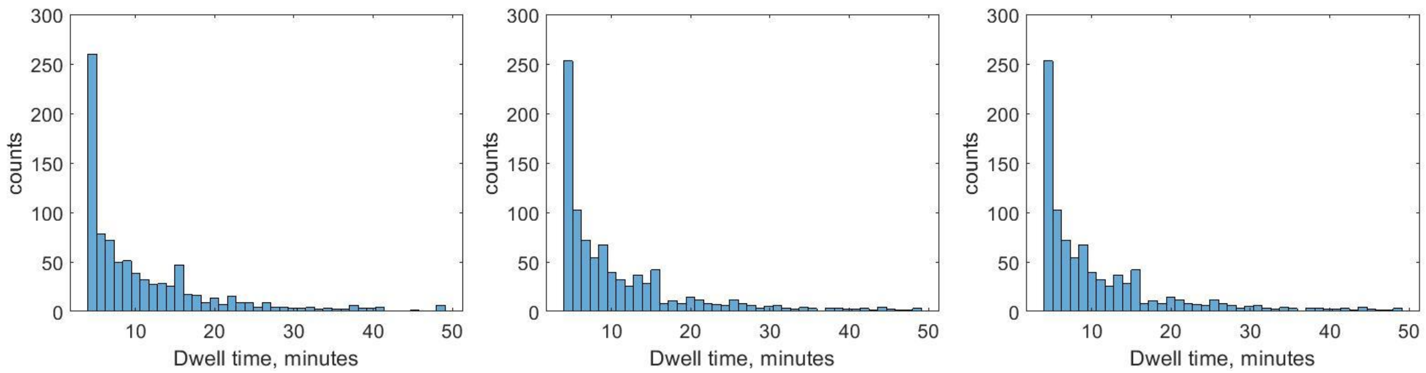

(b)

Figure S9. Cumulative number of binding and debinding events on the 3 replicate spots (on the same sensor surface, inside the same fluidic cell) of miRNA-451 surface probe, with the target GNR-miRNA-451 complex concentration at 100fM (a). The histogram of binding durations ('dwell time') of target particles on the surface for each spot.

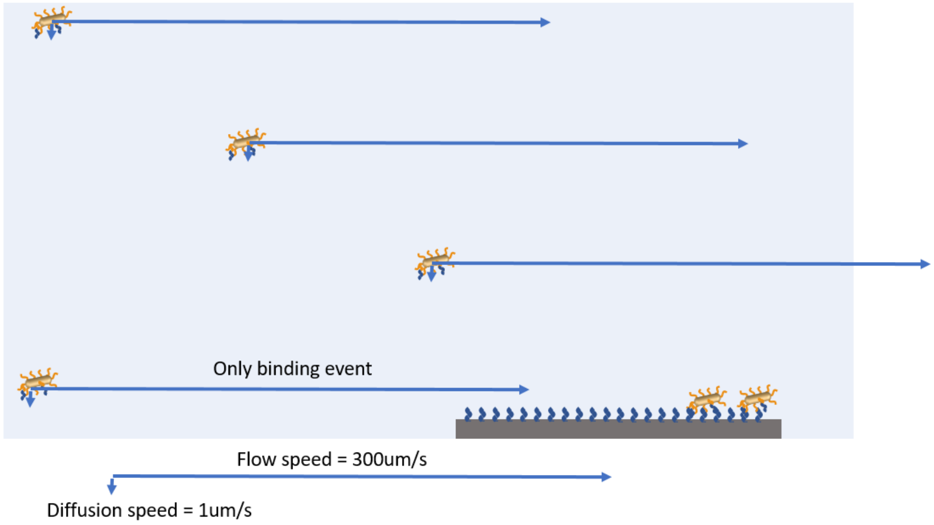

Figure S10. 2D visual representation of the flow cell and sheet density effect

Let us first describe the velocity vectors that describe the movement behavior/direction of particles. In our system there are two components, diffusion and drift (flow).

The diffusion coefficient D is proportional to the squared velocity of the diffusing particles, which depends on the temperature, viscosity of the fluid and the size of the particles according to the Stokes–Einstein relation. In dilute aqueous solutions the diffusion coefficients of most ions are similar and have values that at room temperature are in the range of 1×10^−9 m2/s. For biological molecules the diffusion coefficients normally range from 10^−10 to 10^−11 m2/s, for nanoparticles around 10^-12 m2/s which yields estimated diffusion velocity under 1um/s. The flow/drift velocity under our experimental conditions on the other hand is around 300um/s. Therefore there is a stark contrast between the flow movement and Brownian motion. Given that the intermolecular distance between particles are in the range of hundreds of microns and a particle passes through the entire chip within seconds, the particles that are well above the sensor surface do not have enough time to find the capture probes. Since the capture probes lie on a surface (sensor is a sheet – thickness is infinitely small compared to the volume), the effective concentration our IRIS sensor sees is the sheet density.

Visual representation of the sheet density reduced to 2D provides an easier demonstration, for kinetic flow experiments (Figure S10).

Notice that the particle amount that is in the area (2D case) does not affect the binding, but the particle amount within the linear line interacting with the probes (sensor is a line in the 2D case). The relevant density is the number of particles per unit length of this demonstration. The probability of particles arriving to a capture probe scales with the linear concentration.

Now if we extend the scenario to a 3D case, the sensor expands to a plane and the probability of particles arriving to the surface scales with the amount of particles on the relevant plane (i.e. sheet density), given the kinetic flow conditions (Figure S11).

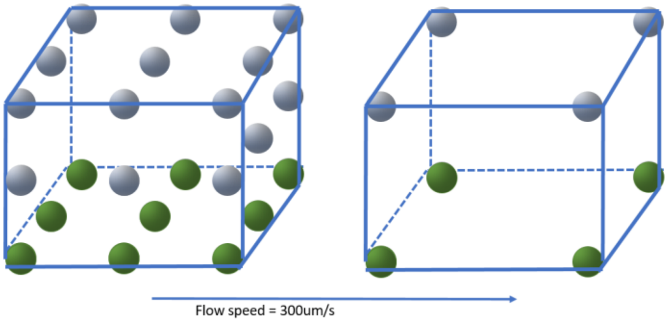

Figure S11. Effective concentration – sheet density, 3D demonstration.

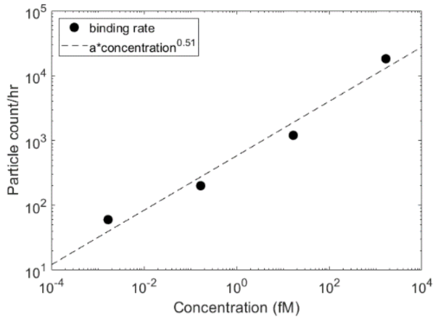

(6)

Figure S12. Dilution experiment performed with AuNPs, showing concentration^0.51 correlation

We support this claim with Figure S6 and Figure S12 and [5], data taken on IRIS systems with similar flow conditions.

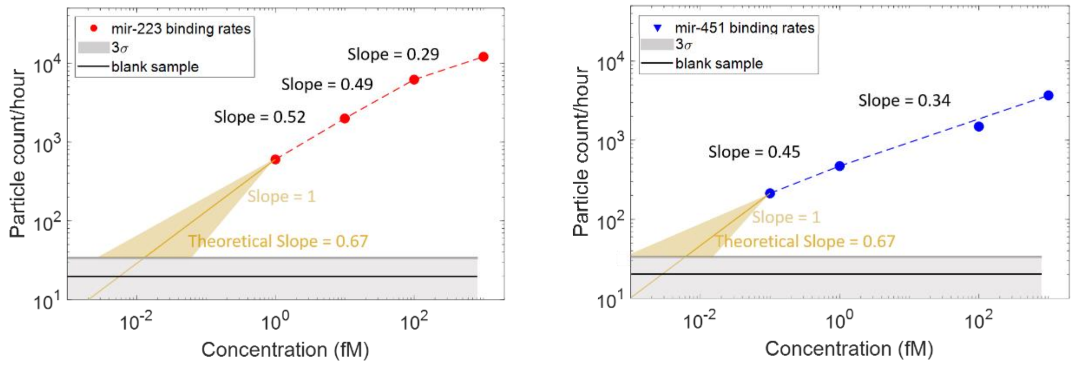

Figure S13. LOD ranges for different extrapolation assumptions: miRNA223 (left), lower bound 1aM, conservative level 13aM, and upper bound 50 aM; miRNA451 (right), lower bound 1aM, conservative level 6.2aM, and upper bound 20 aM.

REFERENCES

[1] A. Yalçın, F. Damin, E. Özkumur, G. di Carlo, B.B. Goldberg, M. Chiari, M.S. Ünlü, A. Yalçin, F. Damin, E. Özkumur, G. di Carlo, B.B. Goldberg, M. Chiari, M.S. Unlü, Direct Observation of Conformation of a Polymeric Coating with Implications in Microarray Applications, Anal. Chem. 81 (2009) 625–630. doi:10.1021/ac801954x.

[2] Lucidant Polymers - Technologies, (n.d.). https://lucidant.com/Technologies (April 15, 2022).

[3] Sevenler, Derin, et al. "Digital microarrays: Single-molecule readout with interferometric detection of plasmonic nanorod labels." ACS nano 12.6 (2018): 5880-5887.

[4] Sevenler, Derin, Jacob Trueb, and M. Selim Ünlü. "Beating the reaction limits of biosensor sensitivity with dynamic tracking of single binding events." Proceedings of the National Academy of Sciences 116.10 (2019): 4129-4134.

[5] Seymour, E., Ünlü, N. L., Carter, E. P., Connor, J. H., & Ünlü, M. S. (2021). Configurable Digital Virus Counter on Robust Universal DNA Chips. ACS sensors, 6(1), 229-237.
